# The feasibility of population screening for paroxysmal atrial fibrillation using hand-held electrocardiogram devices

**DOI:** 10.1093/europace/euae056

**Published:** 2024-02-27

**Authors:** Jonathan Mant, Rakesh N Modi, Peter Charlton, Andrew Dymond, Efthalia Massou, James Brimicombe, Ben Freedman, Simon J Griffin, F D Richard Hobbs, Gregory Y H Lip, Richard J McManus, Kate Williams

**Affiliations:** Primary Care Unit, Department of Public Health and Primary Care, Strangeways Research Laboratory, University of Cambridge, 2 Worts’ Causeway, Cambridge CB1 8RN, UK; Primary Care Unit, Department of Public Health and Primary Care, Strangeways Research Laboratory, University of Cambridge, 2 Worts’ Causeway, Cambridge CB1 8RN, UK; Primary Care Unit, Department of Public Health and Primary Care, Strangeways Research Laboratory, University of Cambridge, 2 Worts’ Causeway, Cambridge CB1 8RN, UK; Primary Care Unit, Department of Public Health and Primary Care, Strangeways Research Laboratory, University of Cambridge, 2 Worts’ Causeway, Cambridge CB1 8RN, UK; Primary Care Unit, Department of Public Health and Primary Care, Strangeways Research Laboratory, University of Cambridge, 2 Worts’ Causeway, Cambridge CB1 8RN, UK; Primary Care Unit, Department of Public Health and Primary Care, Strangeways Research Laboratory, University of Cambridge, 2 Worts’ Causeway, Cambridge CB1 8RN, UK; Heart Research Institute, University of Sydney, Room 3114, Level 3 East, D17 - Charles Perkins Centre, Sydney, NSW 2006, Australia; Primary Care Unit, Department of Public Health and Primary Care, Strangeways Research Laboratory, University of Cambridge, 2 Worts’ Causeway, Cambridge CB1 8RN, UK; MRC Epidemiology Unit, School of Clinical Medicine, University of Cambridge, Cambridge Biomedical Campus, Cambridge CB2 0SL, UK; Nuffield Department of Primary Care Health Sciences, University of Oxford, Radcliffe Observatory Quarter, Woodstock Road, Oxford OX2 6GG, UK; Liverpool Centre for Cardiovascular Science at University of Liverpool, Liverpool John Moores University and Liverpool Heart and Chest Hospital, Liverpool, UK; Danish Center for Health Services Research, Department of Clinical Medicine, Aalborg University, Aalborg, Denmark; Nuffield Department of Primary Care Health Sciences, University of Oxford, Radcliffe Observatory Quarter, Woodstock Road, Oxford OX2 6GG, UK; Primary Care Unit, Department of Public Health and Primary Care, Strangeways Research Laboratory, University of Cambridge, 2 Worts’ Causeway, Cambridge CB1 8RN, UK

## Abstract

**Aims:**

There are few data on the feasibility of population screening for paroxysmal atrial fibrillation (AF) using hand-held electrocardiogram (ECG) devices outside a specialist setting or in people over the age of 75. We investigated the feasibility of screening when conducted without face-to-face contact (‘remote’) or via in-person appointments in primary care and explored impact of age on screening outcomes.

**Methods and results:**

People aged ≥65 years from 13 general practices in England participated in screening during 2019–20. This involved attending a practice nurse appointment (10 practices) or receiving an ECG device by post (three practices). Participants were asked to use a hand-held ECG for 1–4 weeks. Screening outcomes included uptake, quality of ECGs, AF detection rates, and uptake of anticoagulation if AF was detected. Screening was carried out by 2141 (87.5%) of people invited to practice nurse-led screening and by 288 (90.0%) invited to remote screening. At least 56 interpretable ECGs were provided by 98.0% of participants who participated for 3 weeks, with no significant differences by setting or age, except people aged 85 or over (91.1%). Overall, 2.6% (64/2429) screened participants had AF, with detection rising with age (9.2% in people aged 85 or over). A total of 53/64 (82.8%) people with AF commenced anticoagulation. Uptake of anticoagulation did not vary by age.

**Conclusion:**

Population screening for paroxysmal AF is feasible in general practice and without face-to-face contact for all ages over 64 years, including people aged 85 and over.

## Graphical Abstract

**Graphical Abstract:**
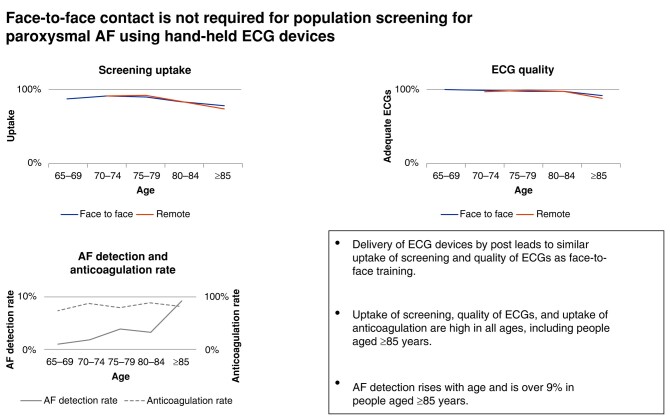

**What’s new?**

- Screening for atrial fibrillation (AF) using hand-held electrocardiogram (ECG) devices has typically involved face-to-face training of people under the age of 80 years with specialist involvement. The purpose of this study was to determine if uptake of screening, quality of ECGs, and uptake of anticoagulation were satisfactory outside a specialist setting and in people of all ages over 65 years.
- Face-to-face contact is not required, as postal delivery results in equally high uptake of screening without loss of ECG quality.
- Uptake of anticoagulation associated with AF screening outside a specialist setting is high and does not decline with the age of the participant, even over the age of 85.
- Atrial fibrillation screening is feasible at all ages, including people over the age of 85, for whom the yield of newly diagnosed AF is high.

## Introduction

Screening for atrial fibrillation (AF) through the use of pulse palpation or single-time point electrocardiograms (ECGs) has become incorporated as standard clinical practice in many countries as a means to reduce stroke,^1,2^ although there is an absence of direct evidence that this leads to clinical benefit.^3^ Indeed, recent trials have not demonstrated that this approach identifies more AF than usual care, in part due to improved AF detection in usual care compared with earlier trials.^4–7^ In recognition that much AF is paroxysmal, there is interest in screening for AF over sustained periods of time,^8,9^ using devices such as hand-held ECGs,^10^ patches,^11,12^ and implantable loop recorders.^13^ While such approaches do detect more AF than usual care,^10,11,13^ uncertainty remains as to whether they lead to the anticipated clinical benefits.^3,14,15^ A preliminary meta-analysis of these trials suggests that more trial data are required to obtain a definitive answer.^16^ A potential advantage of screening using intermittent devices is that the AF that is detected is more likely to be of higher burden than that detected using continuous monitoring,^17^ and burden may be associated with risk of stroke.^18^ Against this back-drop, the Screening for Atrial Fibrillation with ECG to Reduce stroke (SAFER) trial was developed, which involves screening for paroxysmal AF using a hand-held single-lead ECG (Zenicor One, Zenicor Medical Systems AB).^19^ While the feasibility of using this type of device to screen for AF at scale has already been demonstrated,^10^ there is limited experience of using the device in primary care. A similar approach was used in the primary care–based REHEARSE-AF Study, using an AliveCor Kardia hand-held device.^20^ However, duration and intensity of screening (twice weekly over 1 year) were different to what would be feasible on a population scale and therefore envisaged in SAFER (up to four times a day for up to 4 weeks), and the majority of participants were under the age of 75. Furthermore, there was no experience of remote delivery and training in the use of hand-held ECG devices, which became a potentially attractive option as a result of the COVID-19 pandemic, when during lockdowns, face-to-face contact with primary care was discouraged.

There were also design issues to address for population screening for paroxysmal AF. The largest trial of hand-held ECG screening for AF to date was targeted at people aged 75 or 76,^10^ while screening for AF tends to be directed at people aged 65 and over.^2^ We wanted to explore the impact of different ages on agreement to take part, ability to perform ECGs, and AF detection as well as the impact of duration of screening.

Therefore, we carried out studies to assess the feasibility of using hand-held single-lead ECG devices in primary care and without face-to-face contact (‘remote’ delivery) for people of all ages over 64 years and for different durations of screening.

## Methods

Screening for the SAFER feasibility study was carried out in three phases (see *Table 1*). The first two phases involved practice nurse–initiated screening. In the third phase, potential participants were invited to receive the ECG device through the mail. This final phase was incorporated as a direct consequence of the COVID-19 pandemic. In the first two phases, we invited more people to take part in the study than we planned to screen. This was to provide a precise estimate of consent rate to take part in the SAFER trial (where only participants randomized to the intervention arm would be offered screening). In Phase 1, we invited participants to undertake up to 4 weeks of screening and aimed to screen a minimum of 800 participants (160 per practice). Participants were invited for screening in the order in which they returned the consent forms. Some practices were keen to complete screening of all people who gave consent and so continued beyond their target. In order to expedite this, the screening duration was then reduced to 1 week. In Phase 2, we invited participants to engage in 2 weeks of screening, with the same target as Phase 1 of screening 800 participants. In this phase, we did not offer the option of extending screening to more participants. In the final phase, we offered remote screening to all patients who consented to take part in the study.

**Table 1:** Summary of the three phases of the SAFER feasibility study

	Phase 1	Phase 2	Phase 3
Population	4000 people age ≥ 65 years from 5 general practices	4000 people age ≥ 65 years from 5 general practices	825 people age ≥ 70 years from 3 general practices
Time period for screening	March–November 2019	July–December 2019	October 2020–January 2021
Characteristics of screening intervention			
Type of screening	Practice nurse initiated	Practice nurse initiated	No face-to-face contact (‘remote’ screening)
Screening duration and intensity	4 weeks, 4 times/day or 1 week, 4 times/day	2 weeks, 4 times/day	3 weeks, 4 times/day
Summary feasibility outcomes			
Number of patients who gave consent [*N* (%)]	1644 (41.1%)	1803 (45.1%)	320 (38.8%)
Number of participants invited to screening (*N*)	1512	935	320
Number screened [*N* (% of those invited)]	1333 (88.2%) 4 weeks: 986 1 week: 347	808 (86.4%)	288 (90.0%)
Total AF detected	34	20	10
New AF	33	18	10
Known AF	1	2	0
Anticoagulation initiated	27/34 (79.4%)	16/20 (80.0%)	10/10 (100%)
New	27/33	16/18	10/10
Known	0/1	0/2	

AF, atrial fibrillation; N, number; SAFER, Screening for Atrial Fibrillation with ECG to Reduce stroke trial.

### Study population

Participants were aged ≥ 65 years old (except for Phase 3, where the lower age limit was raised to 70 years, as we had by this time determined this would be our lower age limit) and were not coded on the practice system as being on anticoagulation therapy, on the practice palliative care register, or resident in a nursing home. Patients already known to be in AF but not on anticoagulation were eligible, as it has previously been demonstrated that screening such patients provides a useful opportunity to reappraise medical management.^21^ Participating practices ran electronic searches of their medical records to identify eligible patients. A random sample of 800 (275 in Phase 3) eligible patients per practice was drawn from each practice list. The practices then sent these patients an invitation pack that included a participant information sheet (PIS), a covering letter, consent form, reply slip, and Freepost envelope. The PIS and consent form requested access to the patient’s health records and explained that they might subsequently be contacted to participate in screening for AF (which they could decline or accept at that point). A single reminder was sent to non-respondents in Phase 1 and to non-respondents in three of five practices in Phase 2. No reminders were sent in Phase 3 due to time constraints.

### Screening

In the first two phases, participants offered screening were sent a screening leaflet that invited them to attend a screening visit at the practice. At this visit, the practice nurse confirmed verbal consent to take part in screening, showed the participant how to use the Zenicor One single-lead ECG device, and recorded the first ECG. Each recording provides a 30-s trace. The participant was instructed to use the device four times per day and if symptomatic. In Phase 3, participants who indicated that they would accept screening were contacted initially by telephone prior to the device being posted to them, with written instructions on how to use it together with a link to an online video. Telephone support was offered.

ECG traces were not displayed on the device but transmitted via mobile signal to a central secure database, from where they were viewed via a web-based platform. The ECGs were processed using a proprietary algorithm with sensitivity at ECG level against a reference standard of manual interpretation of 97.8%, a specificity of 88.2%, and a positive predictive value of 2.8%.^22^ If multiple ECGs are performed, the sensitivity at patient level will be higher, as only one ECG trace is required to diagnose AF. Those flagged as possible AF by the algorithm were reviewed, and a final diagnosis of AF was made by a cardiologist. Once a diagnosis of AF had been made for a participant, there was no requirement to review all the flagged ECGs. For AF to be diagnosed, the rhythm needed to be present for the full 30 s.

Participants with confirmed AF were invited to attend an appointment with their general practitioner (GP) to discuss the diagnosis, initiation of anticoagulation therapy, and any other appropriate management. If anticoagulation was not initiated, the GP completed a case report form to provide an explanation.

### Outcome measures

Our feasibility outcome measures were consent, uptake of screening, AF diagnosis rate, and uptake of anticoagulation. For a quality of screening outcome, we initially set a target of at least 15 interpretable ECG traces per participant. An interpretable trace was defined as one that was not identified by the proprietary algorithm as being of low quality. Due to very high success rates, we modified this to at least 56 interpretable ECGs over a 3-week period to give greater sensitivity to detect differences by age group or mode of delivery of screening.

### Sample size

For Phases 1 and 2, sending out 8000 invitations enabled estimation of the consent rate with an accuracy of ±1%, screening of ±2%, and detection rate of AF of ±1%. For Phase 3, sending 825 invitations enabled estimation of the consent rate with an accuracy of ±3.5%, screening uptake of ±4%, and proportion of interpretable ECGs of ±3%.

### Analysis

Consent, uptake of screening, and quality of ECG rates are presented by method of screening (practice nurse initiated vs. remote) and by age group (5-year age bands and ≥90). Atrial fibrillation diagnosis rate is presented by duration of screening and age group. Anticoagulation rate is presented by age group. *χ*2 tests for trend are used to examine statistical significance of observed differences by age group, method of screening, and duration of screening as appropriate. For these statistical tests, the oldest age groups are combined. For the key parameters of feasibility, 95% confidence intervals (CIs) were calculated.

### Patient and public involvement

The SAFER programme has a patient and public involvement (PPI) co-investigator, an independent steering committee with a PPI member, and three additional PPI members in the potential target age range for screening. Patient and public involvement members reviewed our patient-facing materials, including information sheets, consent forms, and guidance on how to use the ECG screening device. They advised on how to approach participants and how to inform them about screening procedures.

### Ethical approval

The feasibility studies received ethical approval from the London–Central NHS Research Ethics Committee (18/LO/2066 and 19/LO/1597).

## Results

**Figure 1:**
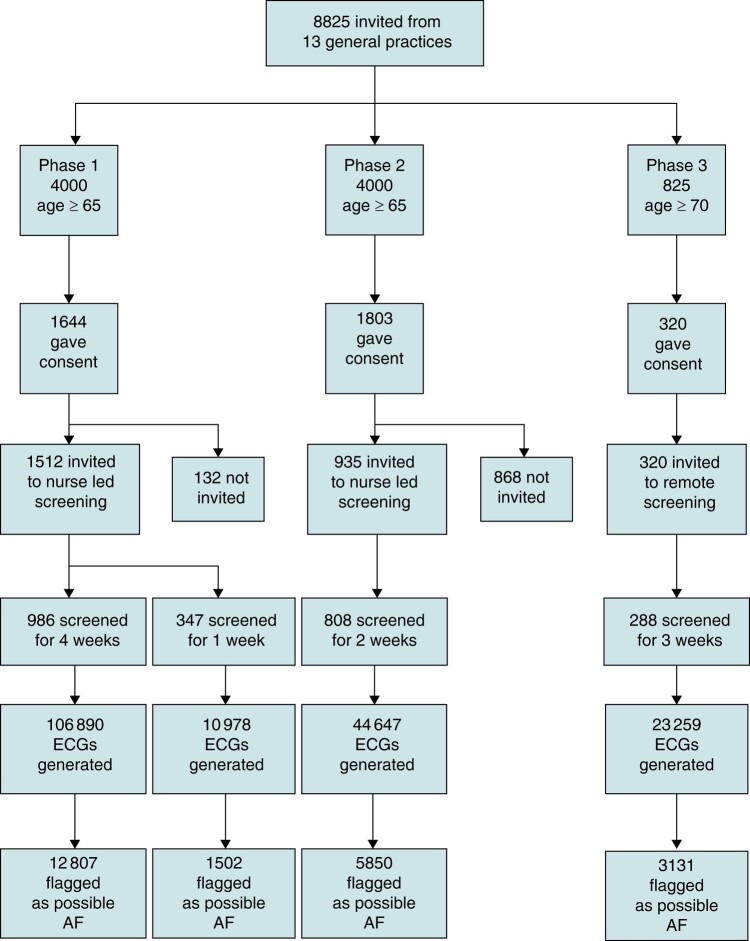
Study flowchart. AF, atrial fibrillation; ECGs: electrocardiograms.

A total of 8825 people were invited to take part in the studies, of whom 3767 (42.7%; 95% CI 41.7–43.7%) agreed to participate (see *Tables 1* and *2* and *Figure 1*). By sex, 43.7% (1805/4131) of men agreed to participate and 41.8% (1961/4693) of women (difference not significant, *P* = 0.075). We invited 2767 people for AF screening, and 2429 (87.8%; 95% CI 86.6–89.0%) completed screening. This generated 185 774 ECGs (mean number per person varying from 31.6 for 1-week screening to 108.4 for 4-week screening). Overall, 23 290 ECGs (12.5%) were flagged as possible AF. There was no difference in completion rates between men and women (1164/1318; 1264/1448), 88.3% vs. 87.3% (*P* = 0.408). Consent rates were higher in practice nurse–initiated screening than in remote screening (43.1% vs. 38.8%, *P* = 0.017). However, if only consent rates from first invitation are considered, then these are similar between practice nurse–initiated and remote screening (3077, 38.5% vs. 320, 38.8%). Uptake of screening was also similar between the two settings (87.5% vs. 90.0%, *P* = 0.198). Consent rate was highest in the age group 70–74 and then declined with age, from 47.8% in 70–74 years old to 23.4% in people aged 85 and over (*P* < 0.001). A similar pattern was observed in screening uptake, with 91.6% of people aged 70–74 completing screening as compared with 77.8% of people aged 85 or over (*P* < 0.001).

**Table 2:** Rate of consent and participation in screening by age group and method of screening (practice nurse initiated vs. remote)

	Practice nurse–initiated screening (Phases 1 + 2)		Remote^a^ screening (Phase 3)		All screening	
	**Consented/invited** ^b^	**Screened/invited** ^c^	**Consented/invited**	**Screened/invited**	**Consented/invited**	**Screened/invited**
Age						
65–69	1112/2434 (45.7%)	677/771 (87.8%)	N/A	N/A	1112/2434 (45.7%)	677/771 (87.8%)
70–74	1155/2386 (48.4%)	711/799 (89.0%)	152/350 (43.4%)	142/152 (92.8%)	1307/2736 (47.8%)	853/951 (89.7%)
75–79	668/1517 (44.0%)	420/470 (89.4%)	92/227 (40.5%)	85/92 (92.4%)	760/1744 (43.6%)	505/562 (89.8%)
80–84	339/926 (36.6%)	232/278 (83.5%)	52/142 (36.6%)	43/52 (82.7%)	391/1068 (36.6%)	275/330 (83.3%)
85–89	130/505 (25.7%)	75/98 (76.5%)	14/61 (23.0%)	14/14 (100%)	144/566 (25.4%)	89/112 (79.5%)
≥90	43/232 (18.5%)	26/31 (83.9%)	10/45 (22.2%)	4/10 (40%)	53/277 (19.1%)	30/41 (73.2%)
≥85	173/737 (23.5%)	101/129 (78.3%)	24/106 (22.6%)	18/24 (75%)	197/843 (23.4%)	119/153 (77.8%)
Sex						
Male	1644/3751 (43.8%)	1018/1157 (88.0%)	161/380 (42.4%)	146/161(90.7%)	1805/4131 (43.7%)	1164/1318 (88.3%)
Female	1803/4249 (42.4%)	1123/1290 (87.1%)	158/444 (35.6%)^d^	141/158 (89.2%)	1961/4693 (41.8%)	1264/1448 (87.3%)
Total	3447/8000 (43.1%)	2141/2447 (87.5%)	320/825 (38.8%)	288/320 (90.0%)	3767/8825 (42.7%)	2429/2767 (87.8%)

N/A, not applicable.

^a^No face-to-face contact.

^b^Numbers represent patients who consented to take part divided by number invited, with % in brackets.

^c^Numbers represent participants who were screened divided by number invited for screening, with % in brackets.

^d^Sex unknown for one participant.

A total of 2407 (99.1%) participants who underwent screening recorded at least 15 interpretable ECGs. Our more stringent criterion (of at least 56 interpretable ECGs) is shown in *Table 3*, which compares the first 3 weeks of screening in people who performed 4 weeks of screening in Phase 1 to the 3 weeks of screening performed in Phase 3. Achievement of this quality criterion was high in both practice nurse–initiated and remote screening (98.3% vs. 97.2%, *P* = 0.257) and high in all age groups (average 98.0%), though dropped in people aged 85 and over to 91.1% (*P* = 0.002). Sex had no significant impact on proportion of interpretable ECGs (97.8% in men vs. 98.3% in women, *P* = 0.491)

**Table 3:** Quality of ECG by age group and method of delivery

Age Sex	Practice nurse–initiated screening (Phases 1 + 2)	Remote^a^ (Phase 3)	Both methods
65–69 all	326/328 (99.4%)	N/A	326/328 (99.4%)
Male	150/150 (100.0%)		150/150 (100.0%)
Female	176/178 (98.9%)		176/178 (98.9%)
70–74 all	339/345 (98.3%)	138/142 (97.2%)	477/487 (97.9%)
Male	168/170 (98.8%)	71/74 (95.9%)	239/244 (98.0%)
Female	171/175 (97.7%)	67/68 (98.5%)	238/243 (97.9%)
75–79 all	190/194 (97.9%)	84/85 (98.8%)	274/279 (98.2%)
Male	94/98 (95.9%)	38/38 (100.0%)	132/136 (97.1%)
Female	96/96 (100.0%)	45/46 (97.8%)^b^	141/142 (99.3%)
80–84 all	79/81 (97.5%)	42/43 (97.7%)	121/124 (97.6%)
Male	41/43 (95.3%)	23/24 (95.8%)	64/67 (95.5%)
Female	38/38 (100.0%)	19/19 (100.0%)	57/57 (100.0%)
85–89 all	29/29 (100.0%)	12/14 (85.7%)	41/43 (95.3%)
Male	16/16 (100.0%)	7/9 (77.8%)	23/25 (92.0%)
Female	13/13 (100.0%)	5/5 (100.0%)	18/18 (100.0%)
≥90 all	6/9 (66.7%)	4/4 (100.0%)	10/13 (76.9%)
Male	3/3 (100.0%)	1/1 (100.0%)	4/4 (100.0%)
Female	3/6 (50.0%)	3/3 (100.0%)	6/9 (66.7%)
≥85 all	35/38 (92.1%)	16/18 (88.9%)	51/56 (91.1%)
Male	19/19 (100.0%)	8/10 (80.0%)	27/29 (93.1%)
Female	16/19 (84.2%)	8/8 (100.0%)	24/27 (88.9%)
Total	969/986 (98.3%)	280/288 (97.2%)	1249/1274 (98.0%)
Male	472/480 (98.3%)	140/146 (95.9%)	612/626 (97.8%)
Female	497/506 (98.2%)	139/141 (98.6%)	636/647 (98.3%)

Numbers represent people who recorded at least 56 interpretable ECGs divided by number screened for at least 3 weeks, with % in brackets.

ECG, electrocardiogram; N/A, not applicable.

^a^No face-to-face contact.

^b^Sex unknown for one participant.

We detected 64 cases of AF through screening (2.6%)—47 men and 17 women (*Table 4*). Detection rate was significantly higher in men than in women (4.0% vs. 1.3%, *P* < 0.001). Three (4.7%) of these were already known to the GP. Rate of AF detection increased with age, from 1.2% in 65–69 years old to 9.2% in people aged 85 and over (*P* < 0.001). One week of screening was associated with a rate of AF detection of only 0.9%, which was not significantly lower than the 3.5–3.6% AF detection observed over 2–4 weeks of screening. Overall, 53 (82.8%) participants found to be in AF were prescribed anticoagulation. Anticoagulation rate did not vary significantly by age (*P* = 0.828) or sex (*P* = 0.419; *Table 5*). None of the three participants already known to be in AF took up the offer of anticoagulation. For 9 of the 11 patients for whom anticoagulation was not commenced, the GP indicated that this was through patient choice. In the other two cases, the decision was made by the GP (not indicated in one as having left atrial appendage occlusion surgery performed; referred to a cardiologist for further investigation in the other).

**Table 4:** Atrial fibrillation detection rate by age group and duration of screening

Age Sex	Duration of screening (weeks)				Any duration
	1	2	3	4	
65–69 all	1/119 (0.8%)	0/230 (0.0%)	N/A	7/328 (2.1%)	8/677 (1.2%)
Male	1/63 (1.6%)	0/96 (0.0%)		4/150 (2.7%)	5/309 (1.6%)
Female	0/56 (0.0%)	0/134 (0.0%)		3/178 (1.7%)	3/368 (0.8%)
70–74 all	1/122 (0.8%)	7/244 (2.8%)	0/142 (0.0%)	8/345 (2.3%)	16/853 (1.9%)
Male	1/60 (1.7%)	5/113 (4.4%)	0/74 (0.0%)	7/170 (4.1%)	13/417 (3.1%)
Female	0/62 (0.0%)	2/131 (1.5%)	0/68 (0.0%)	1/175 (0.6%)	3/436 (0.7%)
75–79 all	1/55 (1.8%)	3/171 (1.8%)	6/85 (7.1%)	10/194 (5.2%)	20/505 (4.0%)
Male	1/32 (3.1%)	1/76 (1.3%)	5/38 (13.2%)	5/98 (5.1%)	12/244 (4.9%)
Female	0/23 0.0%)	2/95 (2.1%)	1/46 (2.2%)	5/96 (5.2%)	8/260 (3.1%)
80–84 all	0/35 (0.0%)	4/116 (3.4%)	3/43 (7.0%)	2/81 (2.5%)	9/275 (3.3%)
Male	0/16 (0.0%)	2/46 (4.3%)	3/24 (12.5%)	2/43 (4.7%)	7/129 (5.4%)
Female	0/19 (0.0%)	2/70 (2.9%)	0/19 (0.0%)	0/38 (0.0%)	2/146 (1.4%)
≥85 all	0/16 (0.0%)	6/47 (12.8%)	1/18 (5.6%)	4/38 (10.5%)	11/119 (9.2%)
Male	0/9 (0.0%)	5/27 (18.5%)	1/10 (10.0%)	4/19 (21.1%)	10/65 (15.4%)
Female	0/7 (0.0%)	1/20 (5.0%)	0/8 (0.0%)	0/19 (0.0%)	1/54 (1.9%)
Total					
Excluding 65–69^a^	2/228 (0.9%)	20/578 (3.5%)	10/288 (3.5%)	24/658 (3.6%)	56/1752 (3.2%)
Male	2/117 (1.7%)	13/262 (5.0%)	9/146 (6.2%)	18/330 (5.5%)	42/855 (4.9%)
Female	0/111 (0.0%)	7/316 (2.2%)	1/141 (0.7%)	6/328 (1.8%)	14/896 (1.6%)
Including all ages	3/347 (0.9%)	20/808 (2.5%)	10/288 (3.5%)	31/986 (3.1%)	64/2429 (2.6%)
Male	3/180 (1.7%)	13/358 (3.6%)	9/146 (6.2%)	22/480 (4.6%)	47/1164 (4.0%)
Female	0/167 (0.0%)	7/450 (1.6%)	1/141 (0.7%)	9/506 (1.8%)	17/1264 (1.3%)

Numbers represent cases of AF divided by number screened, with % in brackets.

N/A, not applicable.

^a^To allow comparison across all duration of screening; sex unknown for one participant.

**Table 5:** Initiation of anticoagulation by age group and sex

Demographic characteristic	AF cases anticoagulated
Sex	
Male	40/47 (85.1%)
Female	13/17 (76.5%)
Age group (years)	
65–69	6/8^a^ (75%)
70–74	14/16^a^ (87.5%)
75–79	16/20 (80%)
80–84	8/9 (88.9%)
≥85	9/11^a^ (81.8%)
Total	53/64 (82.8%)

Numbers represent people anticoagulated divided by number with AF, with % in brackets.

AF, atrial fibrillation.

^a^Includes one person with previously known AF.

## Discussion

We found that population screening for paroxysmal AF in people aged 65 and over using hand-held ECGs is feasible both in primary care and without face-to-face contact (‘remote’ screening). Consent rates associated with the study of remote screening were lower than in primary care, but this is attributable to no reminders being sent to non-respondents in the remote screening study. Uptake of screening was similar in each screening approach. Consent rates were lower in older people, as was uptake of screening in people who had consented to the study (though it was still over 80% in people aged 90 and over). Quality of ECGs was high (98% achieved our quality standard), even in people over the age of 85, in whom 91% achieved the quality standard. Overall detection of AF was 2.6%, rising with age to 9.2% in people aged 85 years and over. In people aged 70 and over, screening for a week resulted in AF detection of 0.9%. Screening for 2, 3, or 4 weeks resulted in 3.5–3.6% AF detection, but this difference from 1 week was not statistically significant. Over 80% of people identified as being in AF were anticoagulated, and this did not vary by age group.

The overall consent rate of 42.7% approximates to what might be expected in a trial, since the information sheet specified that consent would not necessarily result in the offer of screening. As such, it compares favourably with the REHEARSE-AF trial, in which 1272/5846 (21.8%) agreed to participate in a trial of twice weekly screening over the course of a year using a hand-held ECG.^20^ However, our rate was lower than the 51.3% of people who chose to participate in screening in the STROKESTOP trial, but this is not directly comparable, since consent in STROKESTOP was only sought in people who were invited to screening.^10^ In contrast, our uptake of screening in people who gave consent was 87.8%. Therefore, uptake of screening in the SAFER trial can be anticipated to be much higher than that observed in STROKESTOP. Thus, the advantage of the two-stage consent process in SAFER is that the uptake of screening in the future trial should be much higher than in STROKESTOP (predicted 87.8% vs. 51.3%). Therefore, we can anticipate impact of screening to be greater in SAFER than in STROKESTOP. Conversely, from a population perspective, fewer people participated in SAFER—only 37.5% (87.8% of 42.7%) vs. 51.3%. Over 95% of the AF diagnosed in this study was not previously known to the GP. This may reflect increased use of anticoagulants in AF,^23^ which would make such people ineligible for the study, or that people who knew they were in AF did not think taking part would be relevant for them.

This study confirms that screening for paroxysmal AF is feasible in general practice. A new finding is that such screening can be carried out remotely, with no significant drop-off in terms of uptake of screening or quality of resultant ECGs. This is an important observation given the increasing pressures on primary care and the risk of future pandemics. The study also highlights the high yield of new cases of AF in screening people over the age of 85 (over 9%). While it is recognized that some people in this age group may not feel screening is appropriate for them,^24^ for those that do, this study shows that screening can be performed with only minimal loss of ECG quality. Furthermore, uptake of anticoagulation was as high in the very old (≥85 years of age) as it was in younger participants. This discrepancy with data that shows anticoagulation uptake declines with age^23^ may reflect that people who did not want to be anticoagulated are less likely to consent to screening. Thus, if a screening programme was introduced, which would require consent, then similarly high rates of anticoagulation might be anticipated in the very old participants. With regard to duration of screening, <2 weeks is associated with a drop in yield of AF. These findings have been operationalized in the SAFER trial, in which the study population is people aged 70 and over (with no upper age limit), and the intervention is AF screening delivered remotely using a single-lead ECG device over a period of 3 weeks.^19^ In terms of ECG processing workload per person, from the results of this study, such screening might be anticipated to generate a mean of 81 ECGs per person with 11 flagged as possible AF. In terms of numbers of ECGs reviewed by each cardiologist, this will depend upon whether all positive ECGs are reviewed or whether reviewing ceases once a positive diagnosis has been made.

The approach to screening in this study was to identify the target population in terms of a simple criterion—age. More sophisticated approaches to defining the target population are emerging,^25^ for example taking into account co-morbidities, incorporating biomarkers, and analysis of previous sinus rhythm ECGs.^26^ While these approaches hold promise, age remains perhaps the most important single criterion.^27^

### Strengths and weaknesses

The feasibility study was large and involved multiple centres. Estimates around key parameters of feasibility had sufficient precision to inform the trial. Comparisons between remote and practice-based delivery and between different durations of screening were not randomized, so are prone to bias, such as differences in participant risk profile. The remote delivery study took place during the COVID-19 pandemic, and this may have affected participation. Not all flagged ECGs were reviewed by a cardiologist, so it is not possible to report for each patient how many days of screening would have been required. Reading all flagged ECGs may become important in the future given the evidence that AF burden is an important predictor of stroke risk.^18^ Our criterion for ECG quality was relatively crude, so there may have been differences in quality that we did not detect. The proportion of previously known AF may have been under-reported by the GP. Only 37.5% of the total population took part in screening, and non-participants may be at higher risk of stroke.^28^ If screening for AF is to be implemented, strategies will need to be developed to improve uptake in such people.^25^ This was a study of feasibility, not effectiveness, so no conclusions can be drawn as to whether screening for paroxysmal AF should be performed.

## Conclusions

Population screening for paroxysmal AF can be carried out remotely (without face-to-face contact) or via general practice. Participation falls with age, but people over the age of 85 who agree to participate are able to take part in screening nearly as successfully as younger participants. The ongoing SAFER trial (ISRCTN 72104369) will assess whether such screening does reduce stroke incidence compared with usual care.^19^

## Data Availability

Requests for pseudonymized data should be directed to the study co-ordinator (A.D. using SAFER@medschl.cam.ac.uk) and will be considered by the investigators, in accordance with participant consent.
